# TRANSPORTATION AND MOOD: THE ROLE OF TRIP CHARACTERISTICS

**DOI:** 10.1093/geroni/igac059.292

**Published:** 2022-12-20

**Authors:** Holly Dabelko-Schoeny, Noelle Field, Anthony Traver, Ian Murphy, Katie White

**Affiliations:** Ohio State University, Columbus, Ohio, United States; University of Texas -- Arlington, Arlington, Texas, United States; The Ohio State University, Columbus, Ohio, United States; The Ohio State University, Columbus, Ohio, United States; The Ohio State University, Columbus, Ohio, United States

## Abstract

Safe and affordable transportation has a positive impact on the health and well-being of older adults. What is less understood are which factors influence these outcomes. To examine the impact of trip characteristics on the mood of older adults, residents in three neighborhoods in Franklin County, Ohio (n = 32) were provided tablets and used an app (MyAmble) to document their travel. During a 14-day period, 1,190 trips were recorded; 71% of which were completed by car. Participants reported 72% of the trips improved their mood. Perceived importance of the trip, challenges associated with the trip, and trip destinations to social activities and to employment/education explained 33% of the variance in mood. Challenges associated with the trip was the strongest predictor of impact on mood. Identifying trip characteristics that impact mood provides new insights for the design and implementation of travel interventions for older persons.

